# Supplementary material for: Optical heterostructure in a two-dimensional organic crystal
Source: Nat Commun. 2025 Dec 29;17:1168. doi: 10.1038/s41467-025-67937-5 (PMC12858964; doi:10.1038/s41467-025-67937-5)
Supplement: Supplementary file 1 — Supplementary Information [file 41467_2025_67937_MOESM1_ESM.pdf]

Supplementary information

Optical heterostructure in a two-dimensional organic crystal

Kan Liao (廖侃)[1,2,#], Junran Zhang (张军然)[1,#], Xiang-Long Yu (虞祥龙)[3,#], Wenheng Xu (徐文恒)[1], Zhongjing Xia (夏忠静)[1], Dawei Zhou (周大伟)[4], Zilong Mao (毛子龙)[1], Yan Lv (吕焱)[1], Yijun Ming (明义俊)[1], Chao Liu (刘超)[4], Ming Sheng (盛鸣)[4], Kun Liu (刘昆)[1], Zhen Zhang (张震)[2], Chongqin Zhu (朱重钦)[5], Xiaoyong Wang (王晓勇)[2], Chao Zhu (朱超)[4,*], Zhongfu An (安众福)[1,*] and Lin Wang (王琳)[1,*]

[1]State Key Laboratory of Flexible Electronics, School of Flexible Electronics (Future Technologies) & Institute of Advanced Materials, School of Physical and Mathematical Sciences, Nanjing Tech University, Nanjing 211816, China.

[2]National Laboratory of Solid State Microstructures, School of Physics, and Collaborative Innovation Center of Advanced Microstructures, Nanjing University, Nanjing 210093, China.

[3]School of Science, Sun Yat-sen University, Shenzhen 518107, China.

[4]SEU-FEI Nano-Pico Center, Key Laboratory of MEMS of Ministry of Education, School of Integrated Circuits, Southeast University, Nanjing 210096, China.

[5]College of Chemistry, Key Laboratory of Theoretical & Computational Photochemistry of Ministry of Education, Beijing Normal University, Beijing 100875, China.

[#]These authors contributed equally: Kan Liao, Junran Zhang, Xiang-Long Yu.

*Corresponding authors. E-mail: phczhu@seu.edu.cn (Chao Zhu); iamzfan@njtech.edu.cn (Zhongfu An); iamlwang@njtech.edu.cn (Lin Wang)

Table of Contents

Supplementary Figure 1. 1

Supplementary Figure 2 2

Supplementary Note I 3

Supplementary Figure 3 4

Supplementary Figure 4 5

Supplementary Figure 5 6

Supplementary Figure 6 7

Supplementary Figure 7 8

Supplementary Figure 8 9

Supplementary Note II 10

Supplementary Table 1 10

Supplementary Figure 9 11

Supplementary Note III 12

Supplementary Figure 10 14

Supplementary Figure 11 15

Supplementary Note IV 16

Supplementary Figure 12 16

Supplementary Figure 13 17

Supplementary Figure 14 18

Supplementary Figure 15 19

Supplementary Figure 16 20

Supplementary Figure 17 21

Supplementary Note V 22

Supplementary Figure 18 23

Supplementary Figure 19 24

Supplementary Figure 20 25

Supplementary Figure 21 26
Supplementary Note VI 27
Supplementary Figure 22 27
Supplementary Figure 23 28
Supplementary Note VII 29
Supplementary Figure 24 29
References 30

Note: All Supplementary Notes and Figures are arranged in the order of their appearance in the main text to ensure consistency and ease of reference.

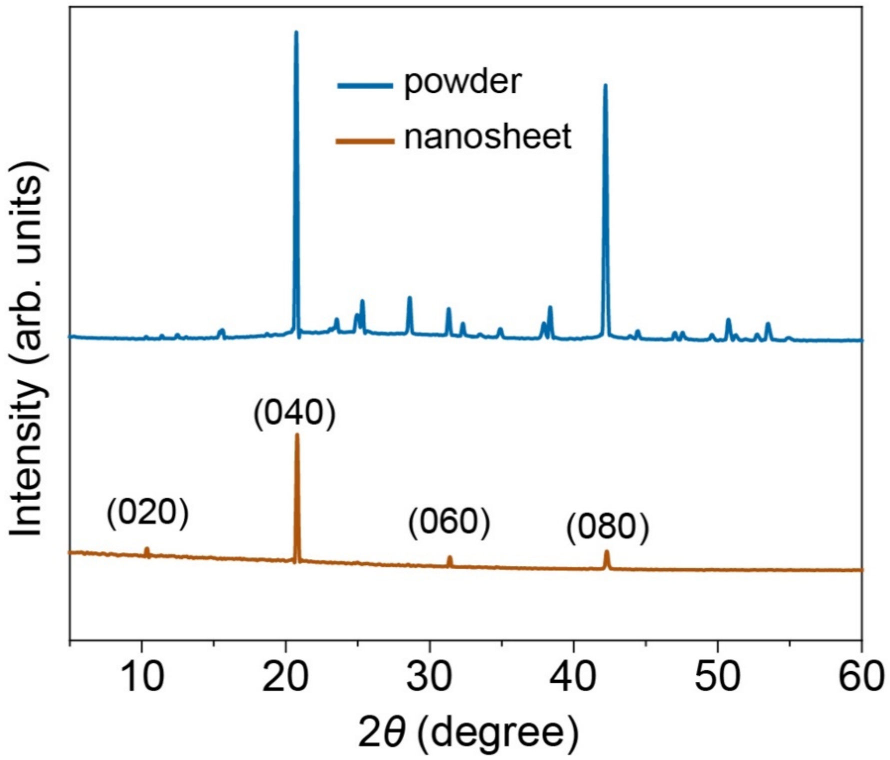

Supplementary Figure 1. XRD patterns of PDBCz powder (blue) and single-crystalline nanosheet exhibiting the optical heterostructure phenomenon (brown). The sharp diffraction peaks of the nanosheet indicate its high crystallinity and preferential orientation. A distinct single (020) diffraction peak confirms that all nanosheets are preferentially oriented along the *b*-axis (vertical direction).

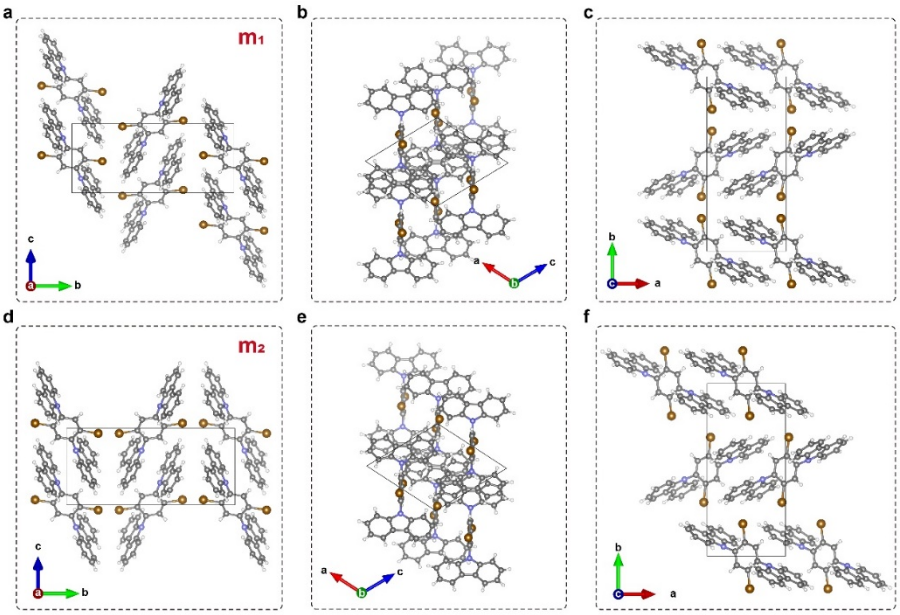

Supplementary Figure 2. Molecular packing structures of the PDBCz unit cell viewed along the *a*-, *b*-, and *c*-crystallographic axes for the m_1 (a-c) and m_2 (d-f) configurations. White, gray, blue, and brown spheres represent hydrogen, carbon, nitrogen, and bromine atoms, respectively. The colored arrows indicate the crystallographic axes (*a*, *b*, and *c*), while the black solid lines outline a single unit cell. The PDBCz crystal belongs to the $P2_1/c$ space group, with lattice parameters of a = 8.710 Å, b = 17.149 Å, c = 8.657 Å, $\alpha = \gamma = 90°$, and $\beta = 116.339°$.

Supplementary Note I. Qualitative comparison of PLQE

(Related to Figure 1e in the main text)

To qualitatively assess the difference in photoluminescence quantum efficiency (PLQE) between the inner and outer zones, we performed a series of spatially resolved and semi-quantitative experiments.

(1) Spatially resolved PL spectroscopy and mapping:

Using a confocal micro-PL system, we separately acquired PL spectra and performed PL mapping for the inner and outer zones (Figure 1d in the main text). The PL intensity in the inner zone is consistently and substantially stronger than that in the outer zone, confirming pronounced emission heterogeneity at the microscale.

(2) Comparison of optical absorption:

To further determine whether the difference in PL intensity originates from variations in absorption, we measured the reflection and transmission spectra of both regions. As shown in Figure 1e of the main text, the two spectra are nearly identical, indicating negligible differences in optical absorption and photon excitation rates between the inner and outer zones[1].

Since the absorption is comparable while the PL emission is markedly stronger in the inner zone, we can qualitatively conclude that its PLQE is significantly higher than that of the outer zone.

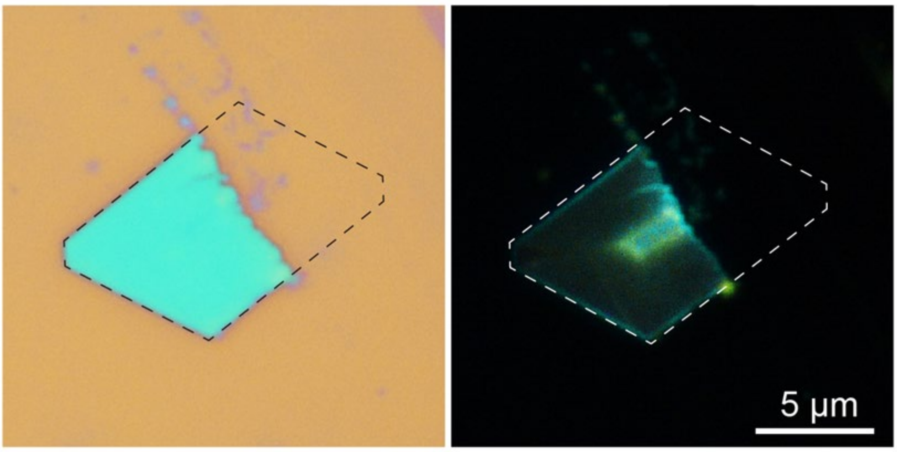

Supplementary Figure 3. Optical (left) and fluorescence (right) images of a half-dissected PDBCz nanosheet. A portion of the nanosheet exhibiting the optical heterostructure phenomenon was mechanically removed using a sharp probe, while the remaining region retained the optical heterostructure phenomenon. This persistence demonstrates that the optical heterostructure phenomenon cannot be attributed to optical microcavity effects, as it remains even after the regular nanosheet geometry is disrupted.

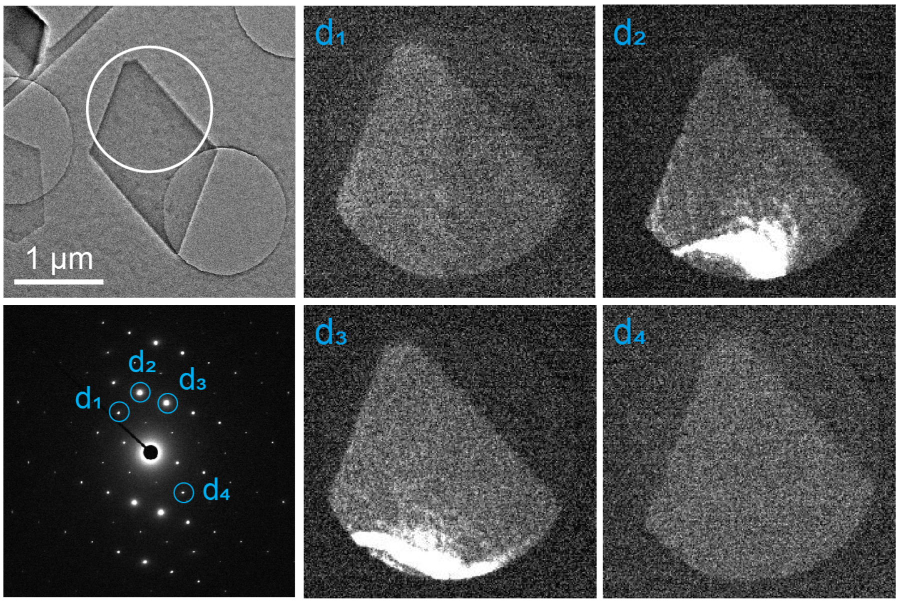

Supplementary Figure 4. Characterization of the structural uniformity and crystallinity of a PDBCz nanosheet. TEM image showing the overall morphology of the nanosheet (top left), and the corresponding selected-area electron diffraction (SAED) pattern confirming its single-crystalline nature (bottom left). Dark-field TEM images obtained using individual diffraction spots (d_1-d_4) reveal uniform crystallinity across the entire nanosheet.

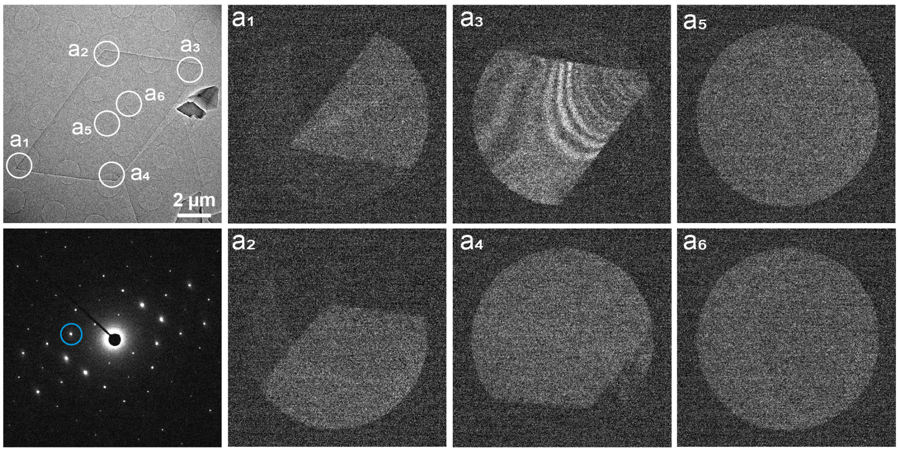

Supplementary Figure 5. TEM, SAED, and corresponding dark-field images of a PDBCz nanosheet. The TEM image (top left) shows the overall morphology, and the SAED pattern (bottom left) reveals the crystalline structure and orientation. Dark-field TEM images (a_1-a_6), obtained by selecting different diffraction spots from distinct regions of the nanosheet, visualize the uniform local crystalline features across the entire sample.

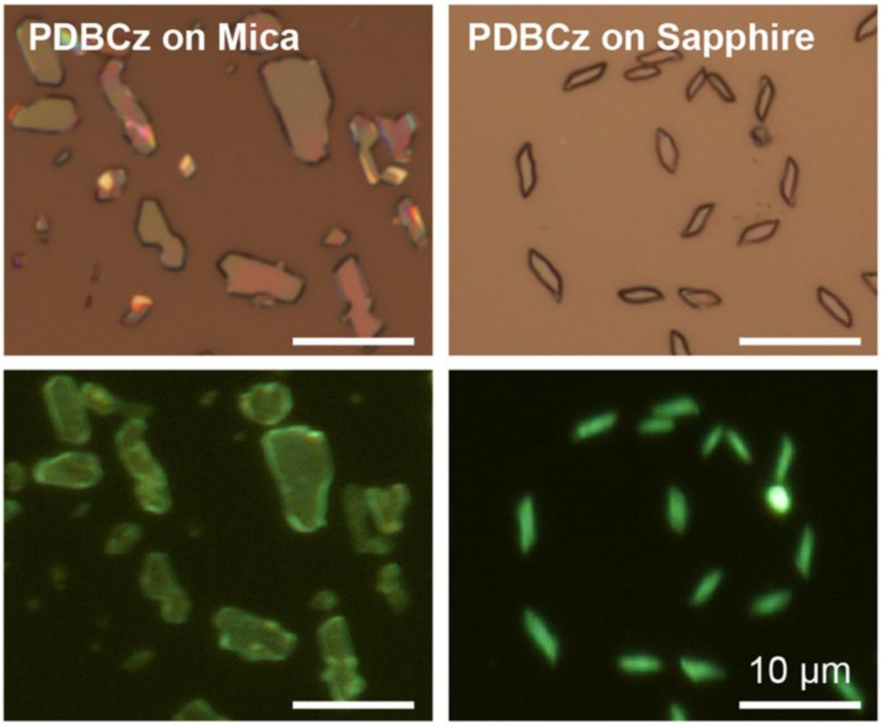

Supplementary Figure 6. Optical (top) and fluorescence (bottom) microscopy images of PDBCz nanosheets synthesized on non-siliceous substrates: mica (left) and sapphire (right). The absence of the optical heterostructure phenomenon in these nanosheets, consistent with that observed on BN substrates, indicates that the occurrence of the optical heterostructure phenomenon is associated with the presence of silicon in the substrate.

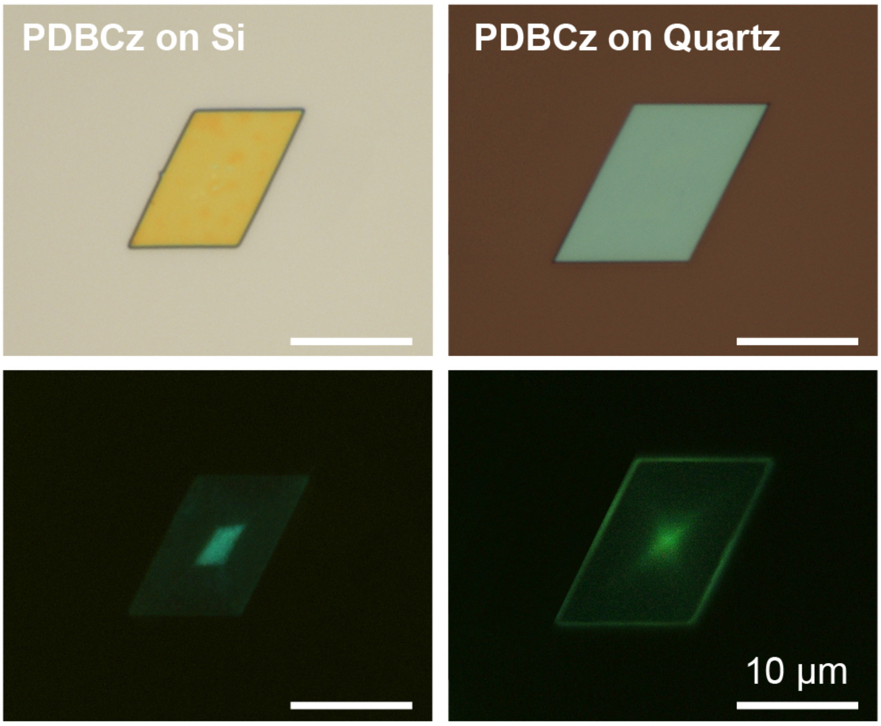

Supplementary Figure 7. Optical (top) and fluorescence (bottom) microscopy images of PDBCz nanosheets synthesized on siliceous substrates: silicon (left) and quartz (right). The presence of the optical heterostructure phenomenon in these nanosheets, consistent with that observed on SiO_2 substrates, further confirms that the occurrence of the optical heterostructure phenomenon is associated with the presence of silicon in the substrate.

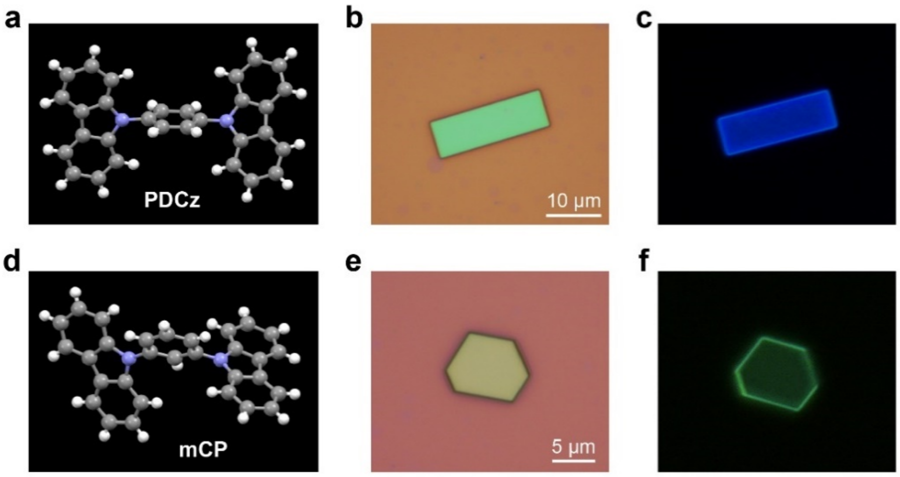

Supplementary Figure 8. Molecular structures (a and d), optical (b and e), and fluorescence (c and f) images of 1,4-di(N-carbazol-9-yl)benzene (referred as PDCz)[2] (top panels) and 1,3-bis(N-carbazolyl)benzene (referred as mCP)[3] (bottom panels) nanosheets on SiO_2/Si substrates. Both molecules share similar backbones with PDBCz but lack bromine (Br) atoms. Specifically, PDCz adopts a para-carbazole configuration, whereas mCP adopts an ortho-carbazole configuration. The absence of the optical heterostructure phenomenon in the fluorescence images of both nanosheets confirms that its occurrence is associated with the presence of bromine.

Supplementary Note II. The calculations of adsorption energy.

(Related to Figures 2g-h in the main text)

Regarding the first-principles calculations, we employed the VASP software, with detailed computational methodologies already described in the Method of main text. For the adsorption energy calculations, we strictly followed the formula:

$$E_{ads}=E_{total}-E_{PDBCz}-E_{sub}$$

where E_{ads} gives the adsorption energy, E_{total} represents the energy of a single PDBCz molecule adsorbed on the substrate, E_{PDBCz} denotes the energy of an isolated PDBCz molecule, and E_{sub} is the energy of the bare substrate. Both pre-adsorption and post-adsorption structures were fully relaxed, with the detailed numerical results presented in the following table.

Supplementary Table 1. The calculated adsorption energy for both SiO_2 and BN substrates.

	PDBCz@SiO_2	PDBCz@BN	PDCz@SiO_2	PDCz@BN
E_{PDBCz}/E_{PDCz} (eV)	-356.231	-356.143	-360.711	-360.711
E_{sub} (eV)	-115.293	-1425.022	-115.293	-1425.022
E_{total} (eV)	-472.288	-1781.166	-476.447	-1785.733
E_{ads} (eV)	-0.764	-0.001	-0.443	0

Regarding the intermolecular interactions between PDBCz molecules, we calculated: 1) The energy of two isolated PDBCz molecules (-359.936 × 2 = -79.872 eV); 2) The energy of two PDBCz molecules interacting with each other (-720.595 eV). The intermolecular interaction energy was then obtained by taking the difference between these values (-0.723 eV).

While regarding the intermolecular interactions between PDCz molecules, we calculated: 1) The energy of two isolated PDCz molecules (-363.863 × 2 = -727.726 eV); 2) The energy of two PDCz molecules interacting with each other (-728.797 eV). The intermolecular interaction energy was then obtained by taking the difference between these values (-1.071 eV).

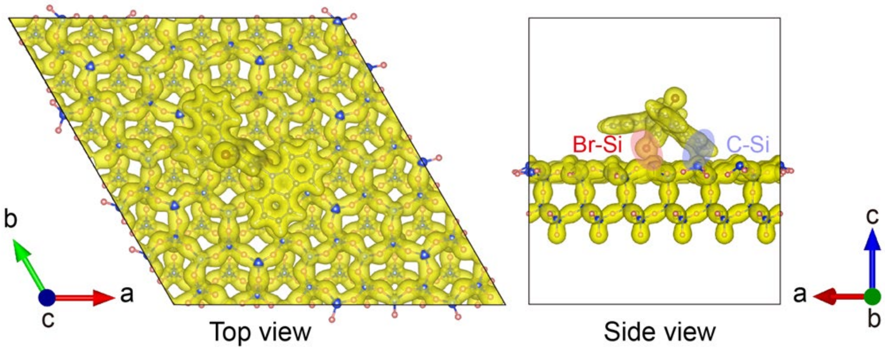

Supplementary Figure 9. Calculated charge density distribution of a PDBCz molecule on the SiO_2 substrate. The isosurface plot shows a pronounced electron density accumulation (in yellow) between the bromine (Br) atom of PDBCz and the silicon (Si) atoms of the substrate, indicating the formation of a covalent bond. This bonding feature is consistent with the theoretical simulations discussed in the main text, confirming the presence of a strong interfacial interaction.

Supplementary Note III. The calculations of surface energy and growth rate.

(Related to Figure 3c in the main text)

The theoretical calculations were performed using *Materials Studio 2017*, where the growth and equilibrium morphologies of PDBCz were evaluated with the Forcite energy method. The simulations employed the universal force field with medium accuracy. Experimentally, monoclinic PDBCz was identified to crystallize in the space group $P2_1/c$, with lattice parameters a = 8.710 Å, b = 17.149 Å, c = 8.657 Å, $\alpha = \gamma$ = 90°, and β = 116.339°, in good agreement with those used in our calculations[4].

As shown in the inset of Figure 3c in the main text, PDBCz nanosheet can adopt two growth orientations on substrates: perpendicular (standing-up) or parallel (lying-down). To elucidate the growth mechanism and packing mode, both growth and equilibrium morphology methods were employed. The growth morphology was analyzed using the attachment energy method, which relates the growth rate of a given surface to the potential energy per unit cell released upon the addition of a new layer in vacuum. This method provides insights into crystal habits under non-equilibrium growth conditions. The attachment energy (E_{att}) is defined as the energy released when a growth slice attaches to the crystal surface[5]. It can be calculated as[6]:

$$E_{att} = E_{latt} - E_{slice}$$

where E_{latt} is lattice energy of the crystal and E_{slice} is the energy of a growth slice with thickness equal to the interplanar distance. The growth rate of a crystal facet is assumed to be proportional to its attachment energy: facets with the lowest E_{att} grow most slowly and thus dominate the morphology. In contrast, the equilibrium morphology method considers that, under equilibrium conditions, crystal habits minimize the total surface free energy[7]. The surface free energy not only governs the crystal growth process but also determines the molecular orientation in the lattice. Indeed, organic molecular packing generally follows the principle of minimizing total surface free energy[8].

As shown in Figure 3c in the main text, our calculations of the (100), (011), and (020) facets reveal that the (020) surface exhibits the lowest surface free energy, corresponding to the slowest growth rate and hence the largest exposed facet. This result is in excellent agreement with experimental observations in our work. The small difference between the surface free energies of the (100) and (011) planes indicates comparable growth rates, reflecting the slight anisotropy in the lattice parameters along

these directions. Overall, the horizontal growth model, with the crystal oriented parallel to the substrate, minimizes the system's surface energy and thus represents the most favorable growth configuration.

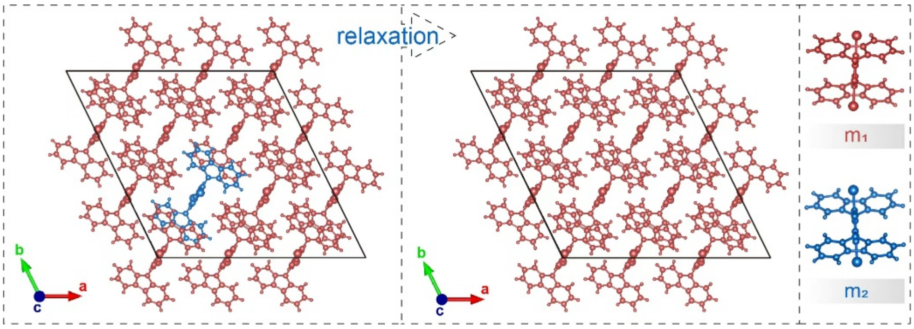

Supplementary Figure 10. Relaxed molecular configurations when m_2 molecules are positioned adjacent to m_1 molecules within the lateral *a*-*c* plane. Structural relaxation drives the system toward a uniform molecular orientation and ordered packing, indicating that heterogeneous m_1-m_2 arrangements in the lateral *a*-*c* plane are energetically unfavorable and spontaneously evolve into a single homogeneous phase.

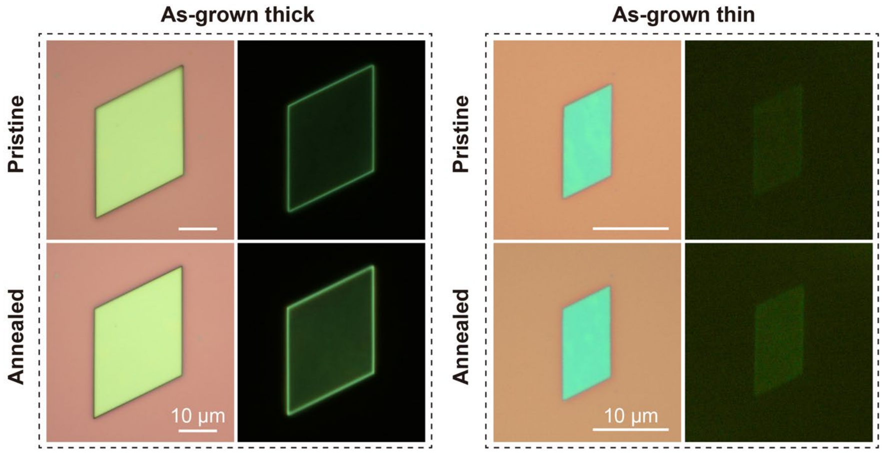

Supplementary Figure 11. Optical (top) and fluorescence (bottom) images of as-grown thick and thin PDBCz nanosheets before and after thermal annealing, both prepared on SiO_2 substrates via the solution method. For both nanosheet types, almost no visible changes were observed in the optical images after annealing, and no optical heterostructure phenomenon was detected in the fluorescence images either before or after treatment. These results indicate that nanosheets grown by the solution method lack the strong interfacial interactions responsible for the optical heterostructure phenomenon, confirming that the occurrence of the optical heterostructure phenomenon is intrinsically linked to strong interfacial coupling.

Supplementary Note IV. Plasma treatment process of the nanosheets.

(Related to Supplementary Figures 13-15)

The nanosheets were thinned using a plasma cleaner (PT-5S Plasma Cleaner). During this process, energetic reactive species generated in the plasma remove organic residues, surface oxides, and micro-particles through a combination of physical bombardment and chemical reactions, while simultaneously activating the surface to enhance hydrophilicity and adhesion.

For processing, the samples were placed in a vacuum chamber evacuated to a low pressure to stabilize plasma generation. Argon was introduced as the working gas, and radio-frequency (RF) or microwave (MW) power was applied to ionize the gas and produce a luminous plasma. The sample surface was exposed to the plasma for a controlled duration (typically a few minutes, depending on the desired thinning level). After treatment, the plasma power was turned off, and the gaseous byproducts were evacuated before the chamber was vented to atmospheric pressure. The processed samples were then retrieved.

A schematic illustration of the plasma treatment setup is provided in Supplementary Figure 12.

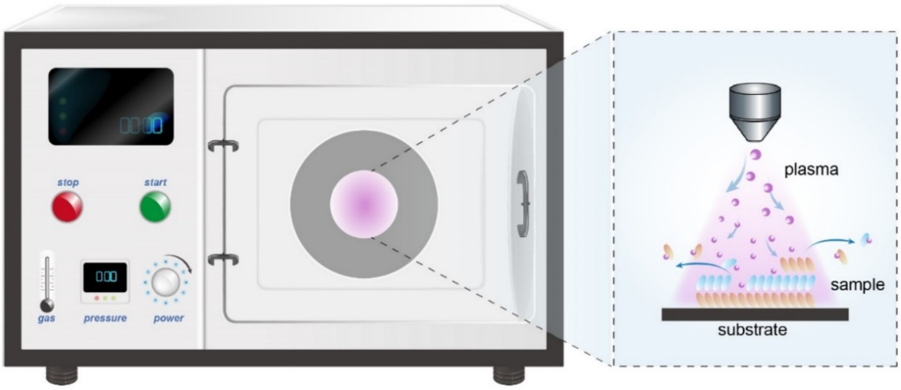

Supplementary Figure 12. Schematic diagram illustrating the plasma treatment process of the nanosheet.

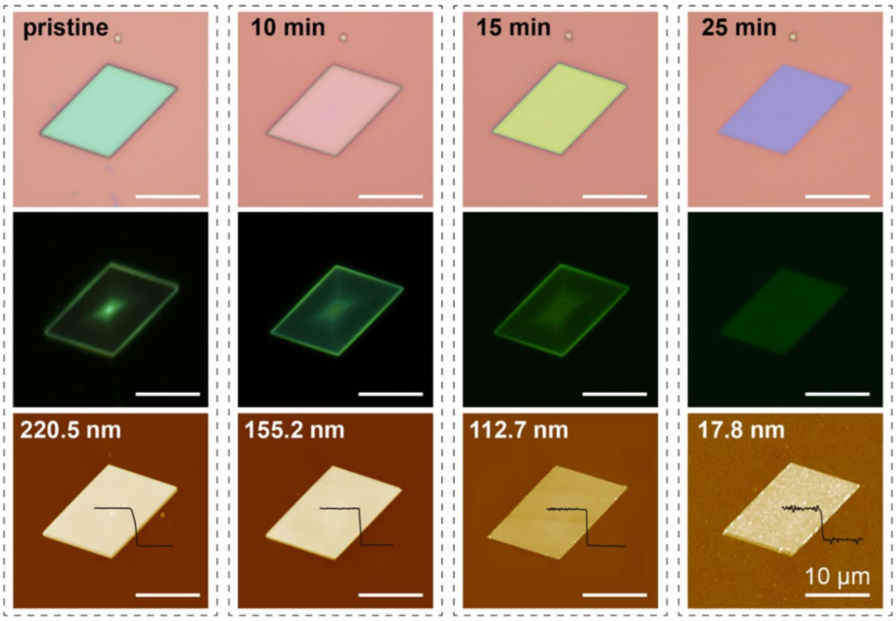

Supplementary Figure 13. Optical (top row), fluorescence (middle row), and AFM (bottom row) images of PDBCz nanosheets subjected to different plasma etching durations (10, 15, and 25 min). The pristine nanosheet, with a thickness of 220.05 nm, exhibits a pronounced optical heterostructure phenomenon. As the plasma etching time increases, the nanosheet thickness gradually decreases, accompanied by a progressive reduction in the luminescence contrast between the inner and outer zones. Notably, when the nanosheet is thinned down to 17.8 nm, the optical heterostructure phenomenon completely vanishes. This observation indicates that the structural transition responsible for the optical heterostructure phenomenon is not limited to the inner zone but occurs predominantly within the top layer of the nanosheet.

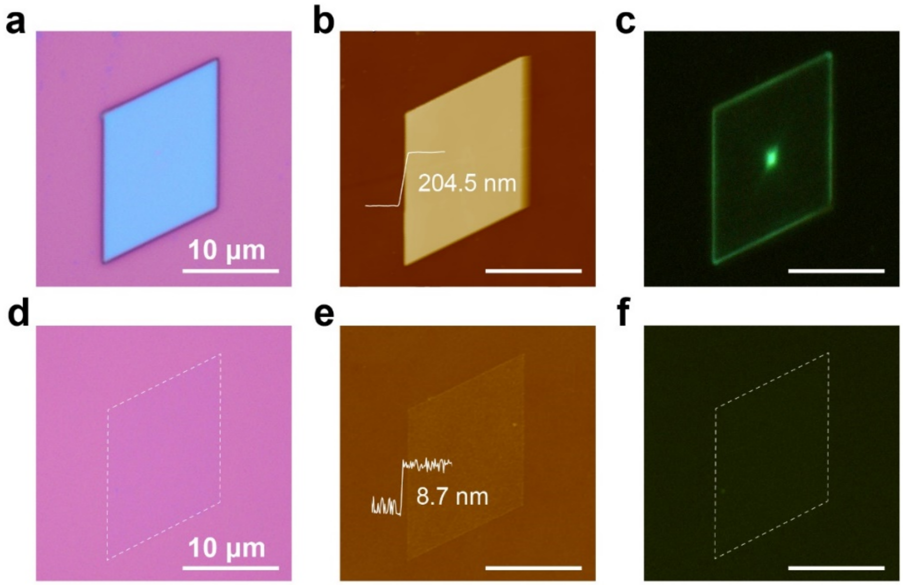

Supplementary Figure 14. Optical (a and d), AFM (b and e) and fluorescence (c and f) images of a PDBCz nanosheet before (top row) and after (bottom row) plasma etching. The pristine nanosheet, with a thickness of 204.5 nm, exhibits a pronounced optical heterostructure phenomenon. After plasma etching, the nanosheet was thinned to 8.7 nm, thinner than the 17.8 nm sample shown in Supplementary Figure 13. As seen in the bottom row, the optical heterostructure phenomenon completely disappears in the etched nanosheet, confirming that plasma treatment effectively removes the upper layer responsible for the optical heterostructure phenomenon.

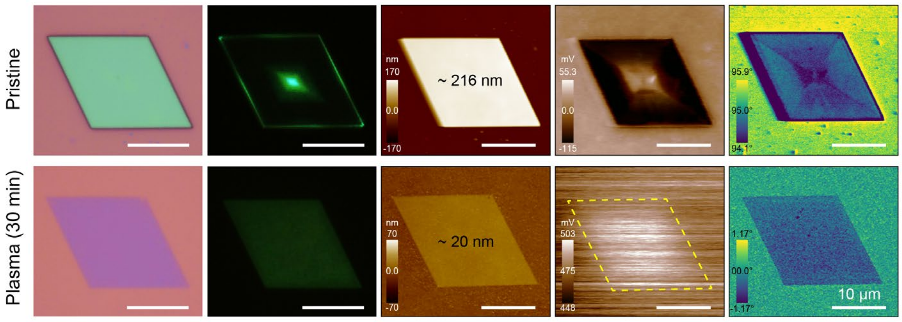

Supplementary Figure 15. Optical, fluorescence, AFM, KPFM, and EFM images (from left to right) of PDBCz nanosheets before and after plasma etching. The pristine nanosheet, with a thickness of ~216 nm, exhibits a pronounced optical heterostructure phenomenon. After 30 minutes of plasma etching, the optical heterostructure phenomenon, as well as the distinct contrast between the inner and outer zones observed in both KPFM and EFM, completely disappears. These results demonstrate that the structural transition responsible for the optical heterostructure phenomenon and the associated KPFM and EFM signal variations is not limited to the inner zone but predominantly occurs within the top layer of the nanosheet.

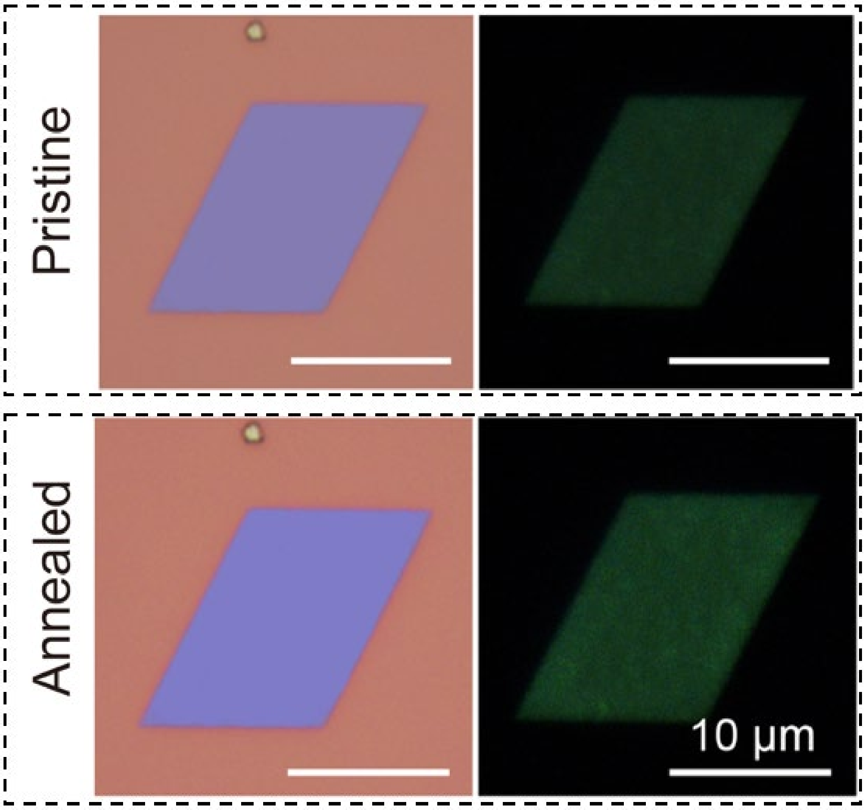

Supplementary Figure 16. Optical and fluorescence images of an artificially thinned PDBCz nanosheet (~18 nm) before and after thermal annealing. No visible damage or change is observed in either the optical or fluorescence images after annealing, indicating the absence of both the optical heterostructure phenomenon and any structural transition in the thin nanosheet.

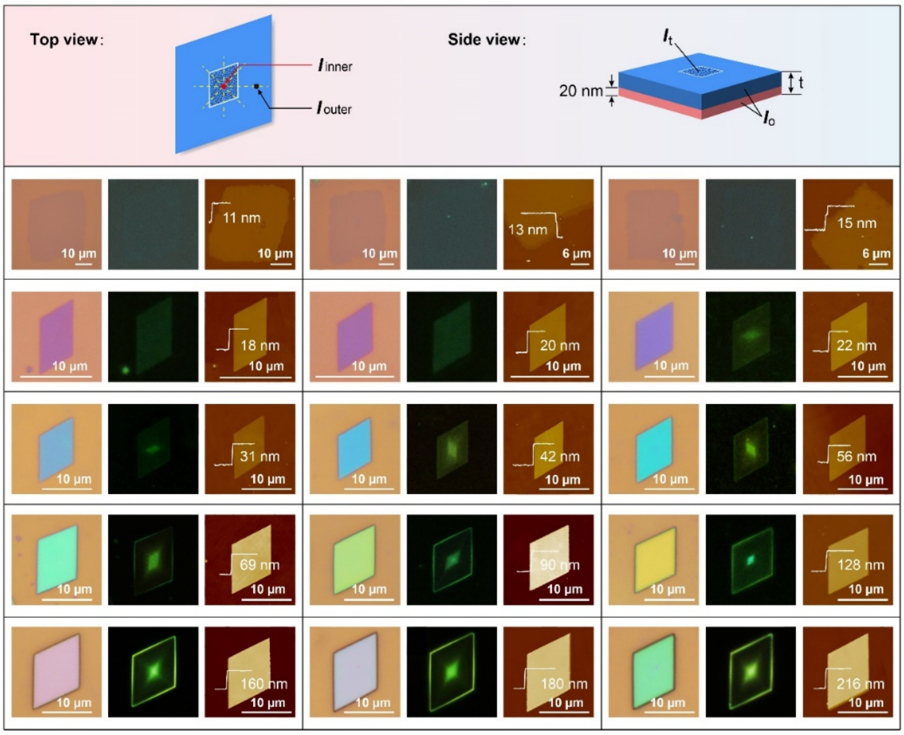

Supplementary Figure 17. Optical, fluorescence, and AFM images of multiple as-grown PDBCz nanosheets with different thicknesses. The nanosheet thicknesses (*t*) were determined by AFM measurements, and the intensity ratio (I_{inner}/I_{outer}) was extracted from the fluorescence brightness. These data form the experimental basis for the I_{inner}/I_{outer} - t relationship plotted in Figure 4c of the main text.

Supplementary Note V. Description of the fitting I_{inner}/I_{outer}.

(Related to Figure 4c in the main text)

In our experiments, the PL spectra of the inner and outer zones were recorded under 405 nm excitation, which can penetrate and excite the bottom layer[9-11]. The excitation spot size was ~1 μm^2, smaller than the inner zone area (~5 μm^2). We define the PL intensity per unit thickness of the bottom layer and the outer zone of the top layer as I_0, and that of the inner zone of the top layer as I_t, which differs from I_0 due to structural transition.

Accordingly, the PL intensity of the inner zone (I_{inner}) can be expressed as:

$$I_{inner} = [I_t \times (t - 20) + I_0 \times 20] \times \text{(excitation spot size)}$$

while that of the outer zone (I_{outer}) is:

$$I_{outer} = (I_0 \times t) \times \text{(excitation spot size)}.$$

Thus, the ratio can be written as :

$$I_{inner}/I_{outer} = [I_t \times (t - 20) + I_0 \times 20]/(I_0 \times t)$$

Using this relation, the experimental data of I_{inner}/I_{outer} as a function of nanosheet thickness (t) were fitted, as shown in Figure 4c of main text. The excellent agreement between experiment and fitting confirms the validity of the model.

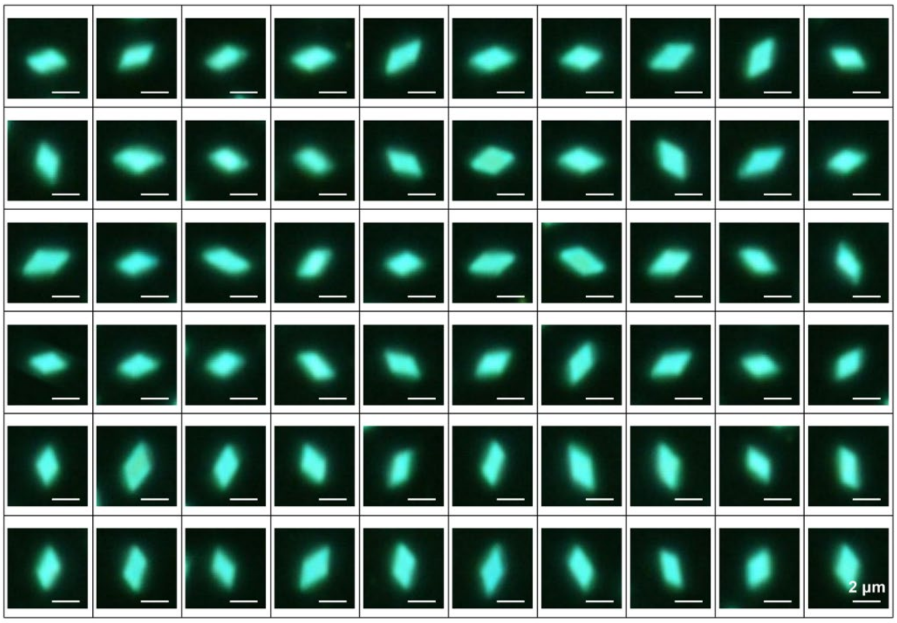

Supplementary Figure 18. Fluorescence images of multiple PDBCz nanosheets captured during the growth stage where the optical heterostructure phenomenon and twin structure begin to emerge. The area of the transition zone in each nanosheet is determined by the overall nanosheet size. All scale bars are 2 μm. Based on our measurements and statistical analysis, the average nanosheet area is approximately 5 μm^2, corresponding to a transition-zone area of about 5 μm^2.

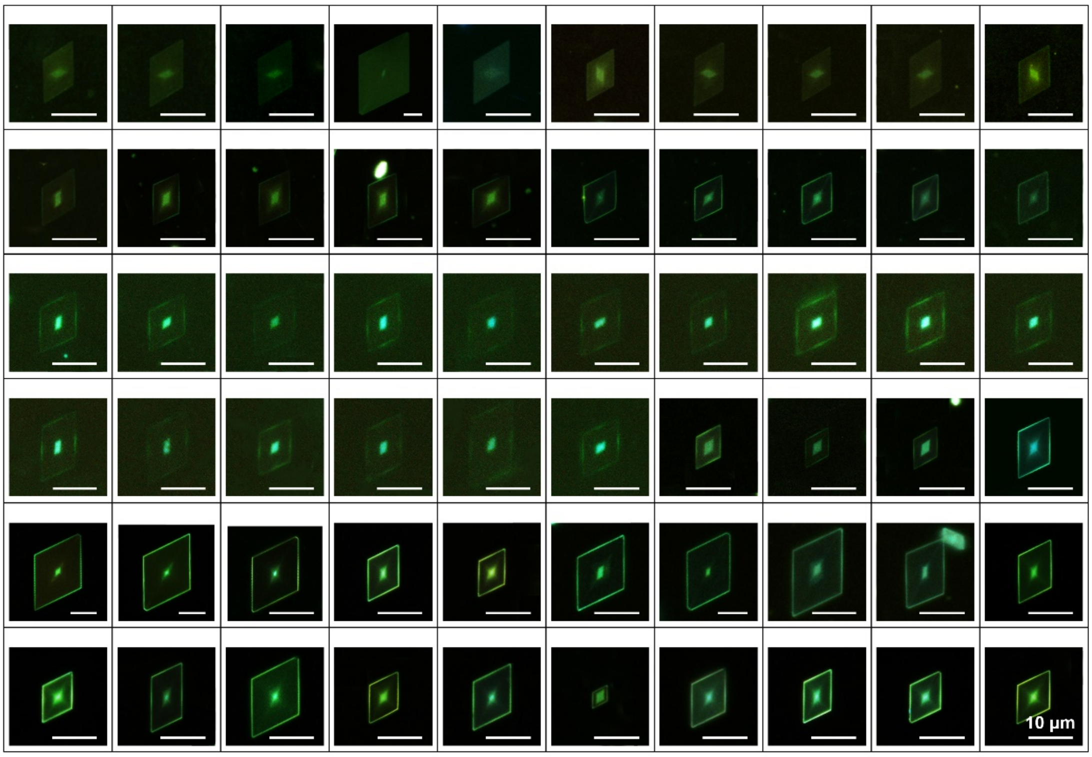

Supplementary Figure 19. Fluorescence images of fully grown PDBCz nanosheets. The area of the inner zone is determined by the size of the central region exhibiting luminescence enhancement. All scale bars are 10 µm. Based on our measurements and calculations, the size of the central luminescent zone in all nanosheets is approximately 5 μm^2, corresponding to an inner zone size of approximately 5 μm^2.

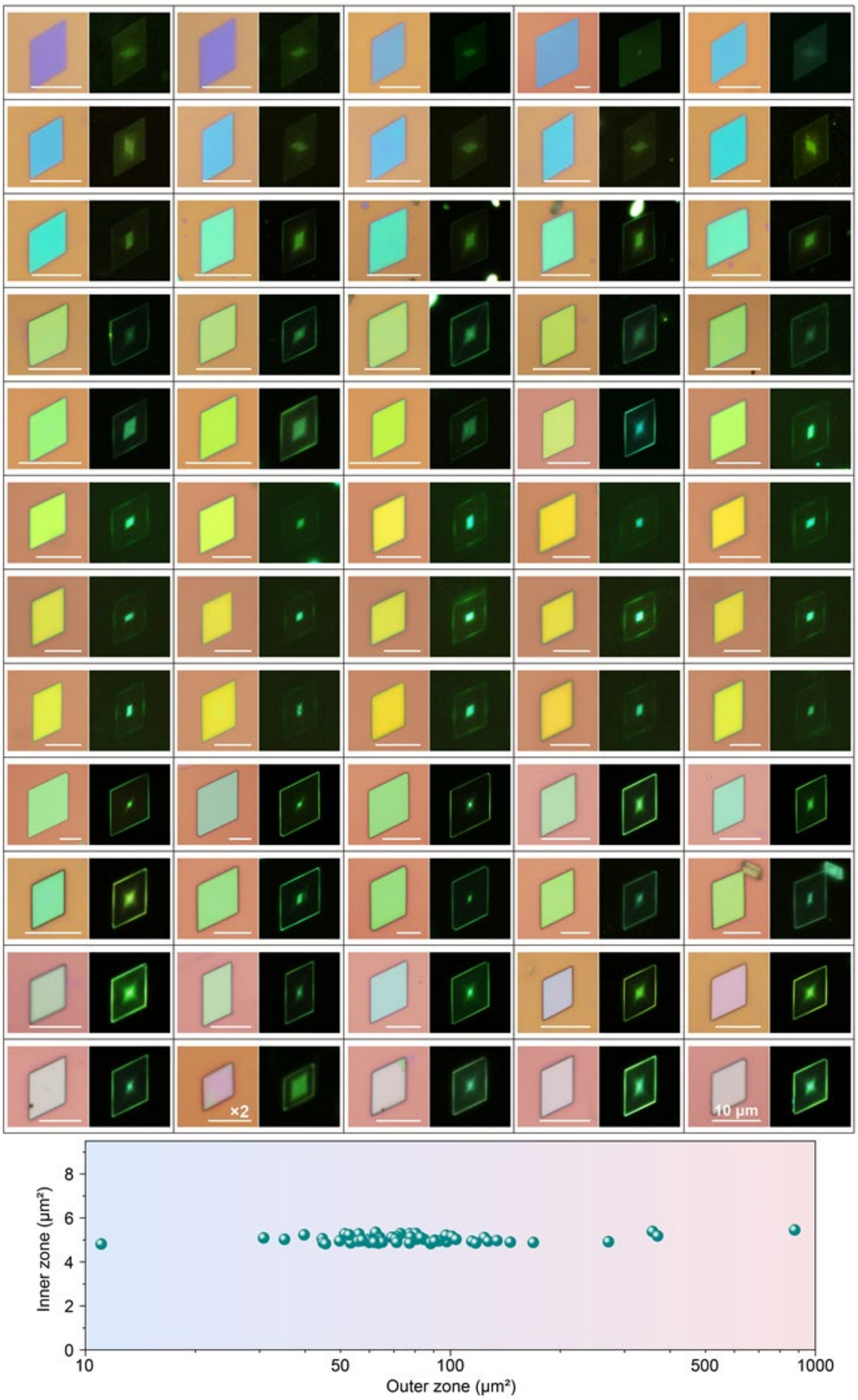

Supplementary Figure 20. Morphology and inner zone consistency of PDBCz nanosheets. Top: Optical and fluorescence images of multiple PDBCz nanosheets with varying outer zone sizes due to different growth times. Bottom: Statistical analysis of the inner zone size as a function of outer zone size, showing that the inner zone dimension remains unchanged with nanosheet growth.

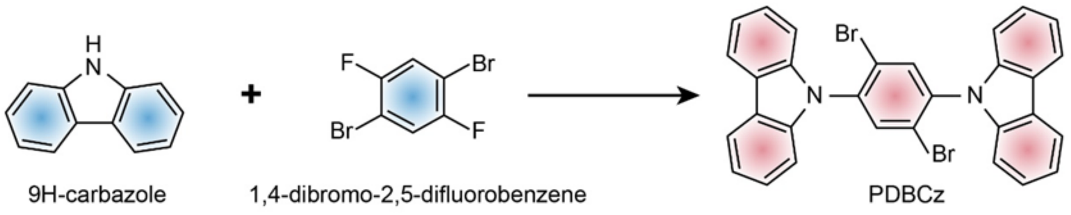

Supplementary Figure 21. Schematic diagram of the PDBCz powder synthesis process.

Supplementary Note VI. The synthesis of PDBCz nanosheets by PVD method.

PDBCz powder (0.1 mg) was loaded into an alumina boat and placed at the center of a tube furnace. The substrate, pretreated with O_2 plasma for 5 minutes to remove surface dangling bonds, was positioned in another alumina boat 10 cm downstream from the furnace center. The source-substrate distance was precisely controlled for each growth to ensure reproducibility. The system was evacuated to a base pressure of 0.1 Pa, followed by the introduction of Ar gas (99.995%) at a flow rate of 100 sccm. The quartz tube was then heated to 310 °C at a rate of 10 °C/min and maintained at this temperature for 1 hour. After growth, the sample was naturally cooled to room temperature. The furnace configuration, including the high- and low-temperature zones, is illustrated in Supplementary Figure 22. For growth on alternative substrates, only minor adjustments to the powder quantity and high-temperature zone settings are required.

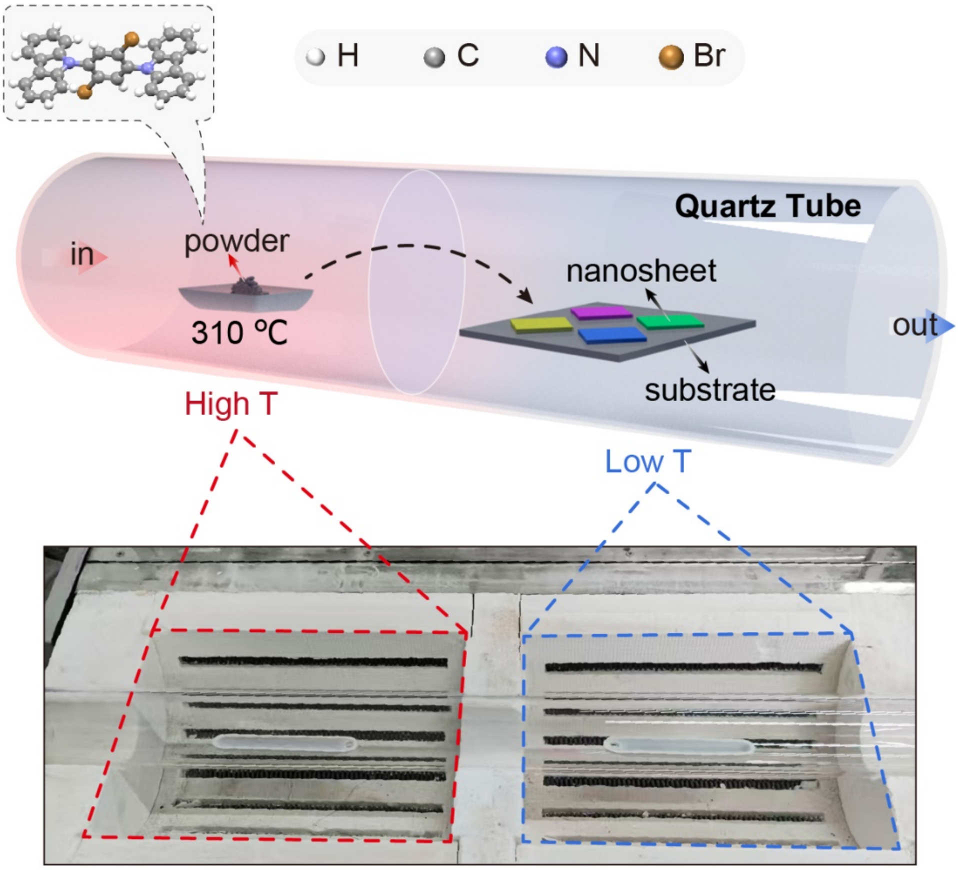

Supplementary Figure 22. Schematic diagram of PDBCz nanosheets grown by physical vapor deposition (PVD) method.

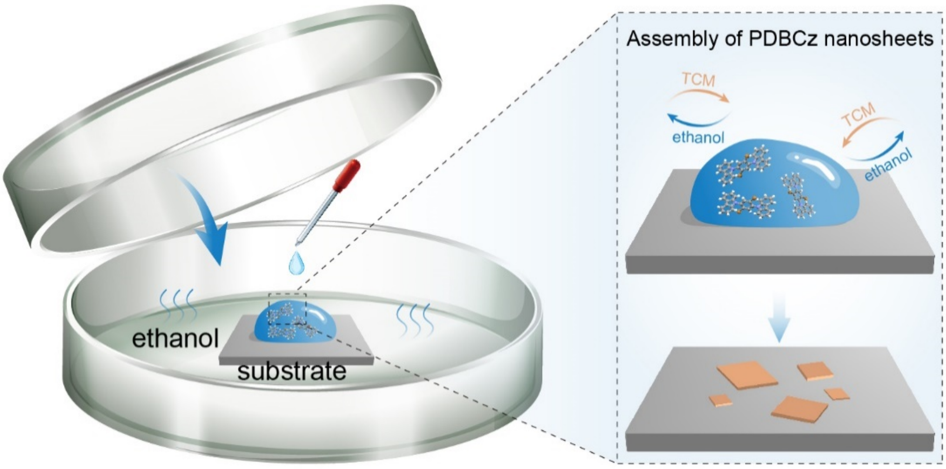

Supplementary Figure 23. Schematic diagram of PDBCz nanosheets grown by solution method.

Supplementary Note VII. Photoluminescence (PL) measurements.

PL spectra from the inner and outer zones of the nanosheets were acquired using a micro-Raman system (Zolix Finder Smart FST2-MPL501-405C1/WITec Alpha 300R). The sample was mounted on a confocal optical microscope, where a 405 nm continuous-wave laser was focused onto the surface through a dry objective lens (NA = 0.55), yielding a ~1 μm spot size. All measurements were performed at room temperature. The PL signal was collected by the same objective, passed through a 405 nm long-pass filter, and directed into a spectrometer equipped with a CCD detector, using a typical integration time of 1 s. The corresponding optical path is illustrated in Supplementary Figure 24. Fluorescence images were recorded using a Nikon DS-Ri2 microscope camera under UV excitation from a mercury lamp (Nikon INTENSILIGHT C-HGFI) with a 330-380 nm band-pass filter.

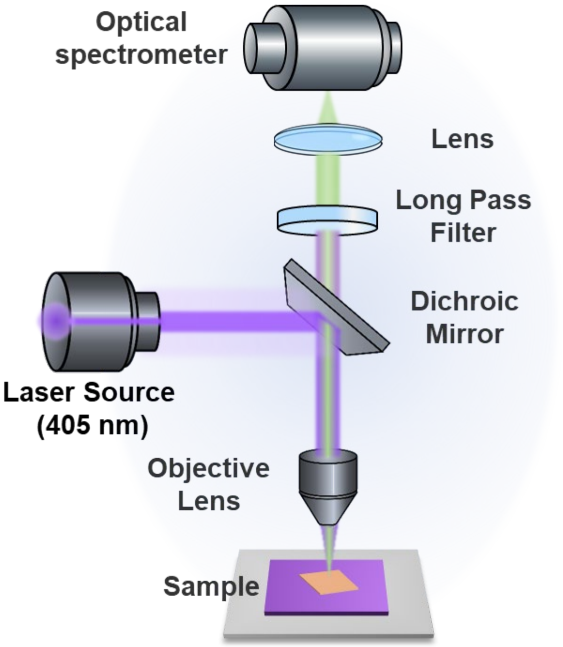

Supplementary Figure 24. Schematic diagram of PL measurement setup.

References

1. Deng, S., et al. Long-range exciton transport and slow annihilation in two-dimensional hybrid perovskites. *Nat. Commun.* **11**, 664 (2020)
2. Shi, H., et al. Highly efficient ultralong organic phosphorescence through intramolecular-space heavy-atom effect. *J. Phys. Chem. Lett.* **10**, 595-600 (2019)
3. Moral, M., Son, W. J., Sancho-Garcia, J. C., Olivier, Y. & Muccioli, L. Cost-effective force field tailored for solid-phase simulations of OLED materials. *J. Chem. Theory Comput.* **11**, 3383-3392 (2015)
4. Liu, K., et al. Tunable microstructures of ultralong organic phosphorescence materials. *Chem. Commun.* **57**, 7276-7279 (2021)
5. Docherty, R., Clydesdale, G., Roberts, K. & Bennema, P. Application of Bravais-Friedel-Donnay-Harker, attachment energy and Ising models to predicting and understanding the morphology of molecular crystals. *J. Phys. D: Appl. Phys.* **24**, 89 (1991)
6. Berkovitch-Yellin, Z. Toward an ab initio derivation of crystal morphology. *J. Am. Chem. Soc.* **107**, 8239-8253 (1985)
7. The Collected Works of J. Willard Gibbs. *Nature* **124**, 119-120 (1929)
8. Li, R., et al. Gibbs-Curie-Wulff theorem in organic materials: a case study on the relationship between surface energy and crystal growth. *Adv. Mater.* **28**, 1697-1702 (2016)
9. Tanabe, I., et al. Electronic excitation spectra of organic semiconductor/ionic liquid interface by electrochemical attenuated total reflectance spectroscopy. *Commun. Chem.* **4**, 88 (2021)
10. Xu, W., et al. Asymmetric charge carrier transfer and transport in planar lead halide perovskite solar cells. *Cell Reports Physical Science* **3**, 5 (2022)
11. de Souza, G. F., et al. Probing the cw-laser-induced fluorescence enhancement in $CsPbBr_3$ nanocrystal thin films: An interplay between photo and thermal activation. *ACS Appl. Mater. Interfaces* **16**, 34303-34312 (2024)
