# Supplementary material for: Optical heterostructure in a two-dimensional organic crystal
Source: Nat Commun. 2025 Dec 29;17:1168. doi: 10.1038/s41467-025-67937-5 (PMC12858964; doi:10.1038/s41467-025-67937-5)
Supplement: Supplementary file 2 — Description of Additional Supplementary Files [file 41467_2025_67937_MOESM2_ESM.pdf]

Description of Additional Supplementary Files

File Name: Supplementary Data 1
Description: Optimized atomic coordinates. This file contains the fractional coordinates of all molecular structures shown in the main-text Figures 1–3 and Supplementary Figures 2, 8, 9 and 10.
